# Large-herbivore nemabiomes: patterns of parasite diversity and sharing

**DOI:** 10.1098/rspb.2021.2702

**Published:** 2022-05-11

**Authors:** Georgia C. Titcomb, Johan Pansu, Matthew C. Hutchinson, Kaia J. Tombak, Christina B. Hansen, Christopher C. M. Baker, Tyler R. Kartzinel, Hillary S. Young, Robert M. Pringle

**Affiliations:** ^1^ Department of Fish, Wildlife, and Conservation Biology, Colorado State University, Fort Collins, CO, USA; ^2^ Department of Ecology, Evolution, and Marine Biology, University of California, Santa Barbara, CA, USA; ^3^ Mpala Research Centre, Nanyuki, Kenya; ^4^ Department of Ecology and Evolutionary Biology, Princeton University, Princeton, NJ, USA; ^5^ ISEM, Université de Montpellier, CNRS, IRD, EPHE, Montpellier, France; ^6^ Department of Anthropology, Hunter College of the City University of New York, New York, NY, USA; ^7^ US Army ERDC Cold Regions Research and Engineering Laboratory, Hanover, NH, USA; ^8^ Department of Ecology, Evolution, and Organismal Biology, Brown University, Providence, RI, USA; ^9^ Institute at Brown for Environment and Society, Brown University, Providence, RI, USA

**Keywords:** cophylogeny, ecological network analysis, generalism and specialism, multiparasitism, phylosymbiosis, wildlife–livestock interface

## Abstract

Amidst global shifts in the distribution and abundance of wildlife and livestock, we have only a rudimentary understanding of ungulate parasite communities and parasite-sharing patterns. We used qPCR and DNA metabarcoding of fecal samples to characterize gastrointestinal nematode (Strongylida) community composition and sharing among 17 sympatric species of wild and domestic large mammalian herbivore in central Kenya. We tested a suite of hypothesis-driven predictions about the role of host traits and phylogenetic relatedness in describing parasite infections. Host species identity explained 27–53% of individual variation in parasite prevalence, richness, community composition and phylogenetic diversity. Host and parasite phylogenies were congruent, host gut morphology predicted parasite community composition and prevalence, and hosts with low evolutionary distinctiveness were centrally positioned in the parasite-sharing network. We found no evidence that host body size, social-group size or feeding height were correlated with parasite composition. Our results highlight the interwoven evolutionary and ecological histories of large herbivores and their gastrointestinal nematodes and suggest that host identity, phylogeny and gut architecture—a phylogenetically conserved trait related to parasite habitat—are the overriding influences on parasite communities. These findings have implications for wildlife management and conservation as wild herbivores are increasingly replaced by livestock.

## 1. Introduction

Parasites account for a large fraction of animal diversity and are key constituents of food webs [1]. They are also pivotal in determining the health, fitness, population dynamics, and community composition of their hosts [2]. Nonetheless, our understanding of parasite diversity remains limited. Owing largely to logistical constraints, many studies focus on single-host/single-parasite interactions, yet most parasites are capable of infecting multiple hosts [3] and most host individuals are infected by diverse parasite assemblages [4]. The relatively few studies of multi-host/multi-parasite networks have revealed fresh insights into parasite transmission and host–parasite coevolution [5], prompting calls for more research on multi-parasitism and consideration of how host biology and environmental factors structure parasite communities [6,7]. These needs are acute given human-induced global changes in host species distributions [8,9] and biodiversity [10]. Particularly important, in terms of both ecological dynamics and human livelihoods, is to understand drivers of cross-species parasite transmission in a world where wildlife are increasingly overlapping with people and their livestock [11].

Historically, sampling limitations have impeded community-level multi-host/multi-parasite analyses. Gastrointestinal nematodes parasitize many different large mammalian herbivore species, yet accurate taxonomic identifications—necessary to quantify diversity and identify host-sharing networks—typically requires retrieving adult specimens from dead hosts or culturing larvae. Moreover, while several databases document host-species–parasite relationships [12,13], data are aggregated over many times and places, obscuring host–parasite sharing patterns among co-occurring individuals. Molecular methods offer a potentially powerful solution to this problem by enabling cheap and efficient sample screening to characterize parasite communities [14,15]. DNA metabarcoding, which involves amplifying and sequencing a short, taxonomically informative genetic region from DNA mixtures (e.g. faeces), is increasingly used in disease ecology [16]. Although applications in parasitology remain nascent [17], they have potential to address questions about multi-parasitism [14,16].

For diverse large-herbivore assemblages, such as those found in African savannahs, such approaches could address knowledge gaps about patterns and determinants of parasite prevalence, diversity and sharing in locations where wild and domestic hosts overlap [18]. Several studies have suggested that parasite richness and community composition are similar among individuals of the same or phylogenetically similar host species [19,20], reflecting the role of host phylogeny in preserving deep coevolutionary linkages between parasites and hosts [21,22] and illuminating the extent to which parasites might switch between and successfully propagate in closely related hosts. However, predictors of parasite richness and composition other than host identity are challenging to disentangle [23,24], due in part to the difficulty of identifying parasites using non-invasive sampling, which is essential for threatened species. Parasite phylogenetic diversity has rarely been investigated, but it has been hypothesized that closely related parasite species occupy similar ecological niches, exhibiting phylogenetic niche conservatism [25]; accordingly, host species with high parasite richness may not necessarily have high parasite phylogenetic diversity, and vice versa.

Three host-specific predictors are often proposed as correlates of parasite species richness across systems and species: body size (and thus parasite habitat size and opportunity to accumulate in large-bodied, long-lived hosts), geographical range size (and thus interspecific transmission opportunities and range of environmental conditions), and population density (and thus intraspecific transmission opportunities) [4,26]. A recent meta-analysis [24] showed that these factors tend to correlate positively with parasite species richness, but that relationships vary depending on whether analyses focus on individuals, populations or species. At the individual level, social-group size may also correlate with parasite richness, as it represents fine-scale intraspecific transmission opportunities [27]. Surprisingly, although transmission in many ungulates occurs via feeding, and many gastrointestinal parasites occupy specific niches within the digestive tract [28], herbivore feeding strategy and digestive strategy are less commonly tested as predictors of parasite community structure; previous work suggests that these factors are related to parasite prevalence and intensity in herbivores [18], and dietary preferences have been linked to parasite composition in other taxa such as birds and fish [29,30].

Because sympatric herbivores share many of the same food and water resources that serve as transmission routes for gastrointestinal parasites [31], studies have constructed parasite-sharing networks (hosts connected to other hosts via shared parasites) using literature records or morphological identifications [18,32]. Such networks are useful because node-specific metrics can be used to identify hosts that are central in various ways, such as (a) those having many links to other species via their parasites (high degree); (b) those sharing parasites with many other hosts (high closeness centrality); (c) those sharing parasites with distinct groups, acting as bridges (high betweenness centrality); and (d) those sharing parasites with other well-connected hosts, thereby occupying core positions (high eigenvector centrality). Central hosts may be likely to affect transmission dynamics for many other species. Likewise, bipartite host–parasite networks can illuminate potential parasite coextinctions when host species are lost, which should be greatest for hosts with many unique parasite links [33].

We used DNA metabarcoding of the ITS-2 region to analyse gastrointestinal nematode DNA (Strongylida) in fecal samples from a diverse community of wild and domestic large herbivores (greater than or equal to 5 kg) in an East African rangeland. Strongylida are among the most common metazoan parasite groups in large herbivores and infect various parts of the digestive tract, including the stomach and intestines. While taxonomic reference data for nematodes remain limited, DNA metabarcoding enables comparative analysis of diversity and composition by clustering sequences by similarity even when taxonomic names are lacking, as has been validated for both parasitic and free-living nematodes [34,35] (see also [36,37]). We addressed three questions about the diversity, composition and sharing patterns of Strongylid (henceforth ‘parasite’) communities in this diverse assemblage.

First, to what extent can host characteristics predict total parasite prevalence (i.e. proportion of hosts infected by at least 1 molecular operational taxonomic unit (mOTU)), richness and phylogenetic diversity? We hypothesized that, in addition to strong effects of host species identity on parasite assemblages [19], hosts that feed on understorey plants would have higher parasite richness and prevalence than overstorey feeders, because the former are more likely to consume infective parasite larvae and eggs [32,38]. Because foregut fermenters (here, 12 ruminants and 2 pseudoruminants) have more complex guts that may provide a wider range of parasite niches [28], we predicted that parasite diversity would be higher in those species compared to hindgut fermenters. We likewise hypothesized that after accounting for host relatedness, total prevalence, richness, and phylogenetic diversity would increase with host body size (which is correlated with home-range size) and social-group size. Second, which factors explain dissimilarities in parasite community composition among herbivores? We expected that the same host traits predicted to influence parasite prevalence and diversity would structure community composition, with strong effects of host species identity, and that host and parasite phylogenies would be congruent, consistent with host–parasite coevolution [22]. Third, to what extent are parasite mOTUs shared among hosts, and which host species play key roles in a parasite-sharing network? We expected host–parasite linkages to have an aggregated distribution (i.e. that most parasites specialize on only one or two host species and few generalize across many different hosts, as is typical in host–parasite networks [39]). We further expected that a host–host network (with host species connected by shared parasites) would link closely related hosts, including both domestic and wild species, and that network centrality would increase with decreasing evolutionary distinctiveness [40].

## 2. Material and methods

Mpala Research Centre and Conservancy (0°17′ N, 37°52′ E) comprises 200 km^2^ of semi-arid thorn-scrub savannah managed for wildlife conservation and livestock production [41,42]. The large-herbivore assemblage includes two-dozen wild species and 5 livestock species (in 2021, approximately 1300 cattle, approximately 300 sheep and goats, approximately 130 camels, and some donkeys).

### (a) DNA sequencing and data processing

Detailed DNA extraction, qPCR, PCR and metabarcoding, and bioinformatic methods are in Appendix I; we provide a summary below. Prevalence was assessed using qPCR on 550 fecal samples; community metrics were assessed on 281 of these samples that contained strongylid DNA. Samples were collected during six sampling bouts over five years (2013–2017), including a substantial subset of those analysed for plant and bacterial DNA in [43] and a set of zebra samples analysed in [35]. Samples were broadly dispersed and interspersed in space and time [43]; individual identities are unknown, but the spatio-temporal breadth and low ratio of sample sizes to species' abundances [44] means that duplicate samples from a given individual are highly unlikely. These samples represent 20 large-herbivore species (electronic supplementary material, Appendix I: table S1), which together account for approximately 95% of individuals in the community [44], span orders of magnitude in body mass (5–5000 kg), and differ in digestive system (foregut/hindgut fermentation), diet (1%–97% grass [45]), social behaviour (solitary/gregarious), evolutionary history (3 orders, 7 families, 15 wild and 5 domesticated species) and conservation status (2 endangered, 2 vulnerable, 2 near threatened, 9 least concern [46]). Among domesticated species, sheep and goats are regularly treated with anthelminthics (albendazole, Endotape), cattle are treated at weaning and occasionally thereafter, and camels and donkeys are not routinely treated (D. Hewett, Mpala Ranch manager, 2021, personal communication).

We performed duplicate qPCR on all 550 DNA extracts to detect Strongylida DNA (NC1/NC2 primers for ITS-2 gene; [47]). As a sensitivity check, we compared qPCR results to fecal egg counts for 37 impala and warthog samples sequenced in this run; we detected eggs in 35/37 qPCR-positive samples, and cycle threshold (*C*_t_) values were correlated with egg-counts (*ρ* = −0.51, *p* = 0.001). Samples with mean *C*_t_ less than 35 were considered positive for parasite DNA; this threshold corresponded to approximately 20 eggs per gram and fell below the detection threshold of the Modified McMaster technique (40–50 eggs per gram) [48]. We focused sequencing effort on a subset of qPCR-positive samples (*n* = 323), which we re-amplified using dual-indexed primers, multiplexed, purified and sequenced together with 502 samples from another study along with positive, negative and extraction controls (Illumina MiSeq: 2 × 300 bp, 24 M reads).

Sequences were demultiplexed, filtered and clustered into mOTUs at both 98% and 99% similarity (using OBITools [49]). We used the nemabiome database (https://www.nemabiome.ca/) [50] for taxonomic assignments. Inferences about diversity and network structure may be sensitive to the threshold used to cluster mOTUs; we present results from the 98% similarity threshold in the main text [35], but results were qualitatively similar using the 99% threshold (electronic supplementary material, Appendix II). We filtered the sample-by-mOTU table to account for any low-abundance reads in negative controls (*microDecon* package [51]), dropped samples with fewer than 1000 reads, excluded mOTUs with less than 2% relative read abundance (RRA) per sample, and rarefied to 748 reads, the minimum number in any sample (validation of this approach is in electronic supplementary material, appendix I; see also [52]). We excluded one sample that was probably erroneously identified and one that lacked metadata (electronic supplementary material, appendix I). All sheep, all goat and the two waterbuck samples were qPCR-negative and were excluded from subsequent analyses. Anthelminthic treatment in cattle probably altered their parasite communities, so we excluded them from analyses of species' traits on parasite patterns but retained them in all other analyses.

### (b) Statistical analyses

Analyses were performed in R v. 4.1.1 [53].

#### (i) Parasite mOTU prevalence, richness and phylogenetic diversity

We determined parasite richness by summing the number of mOTUs detected in each sample. We calculated Faith's phylogenetic diversity (PD) with the *pd* function in *picante* [54], using a phylogenetic tree of parasite mOTUs generated from metabarcoding data (electronic supplementary material, figure S10)*.* Because taxonomic richness correlates with phylogenetic diversity, we also calculated standardized PD (*ses*PD) to determine if phylogenetic diversity in certain samples differed from expectations while controlling for mOTU richness. To avoid biases [55], we excluded the root of the phylogeny, resulting in exclusion of 12 samples with one parasite mOTU (*n* = 245 for PD and *ses*PD analyses after also omitting cattle).

We used two sets of models to analyse the effects of host species identity, traits and phylogeny on prevalence, richness and phylogenetic diversity. First, we used GLMs (with Binomial, Poisson and Gaussian error structures for prevalence, richness and phylogenetic diversity, respectively) to determine the proportion of variance described by host species identity. Second, we tested whether log-transformed body mass, proportion of understorey vegetation in the diet (for each sample), and digestion type (foregut/hindgut) predicted parasite prevalence, richness and phylogenetic diversity using GLMMs (*MCMCglmm* [8]) with random effects for host species and sampling bout. We ran models with and without controlling for host phylogeny [56]. We did not include range size because it was highly correlated with body mass (*ρ* = 0.89, *p* < 0.001).

We used body size measurements from PanTHERIA [57]. Group sizes and home range sizes were obtained from publications from our study site whenever possible, or otherwise from PanTHERIA or by using averages from a literature search (details in electronic supplementary material, table S2). To calculate understorey diet proportion for each sample, we summed the RRA of plant mOTUs less than 2 m tall for individual fecal samples based on diet data in [43] and local plant-height data [42]; for 60 samples not present in the diet dataset, we used the host's species-wide mean.

#### (ii) Host characteristics and parasite community composition

To visualize patterns of parasite community composition among host species, we ordinated Bray–Curtis dissimilarities calculated from the sample-by-mOTU matrix using non-metric multidimensional scaling (NMDS; *k* = 3 dimensions) in *vegan* [58]. We compared parasite community composition among host species using perMANOVA (*adonis2* function). To evaluate temporal variation, we analysed the effect of sampling period for a subset of six broadly sampled species (*n* ≥ 5 samples for at least 2 periods), first by comparing period and host identity when considering all species together and then by testing the effect of period for each species. To account for phylogenetic similarity among mOTUs, we repeated analyses using weighted UniFrac distances [59] of the same 281 samples, including cattle (*phyloseq* [60]).

To investigate drivers of taxonomic and phylogenetic parasite community composition, we created a host species-by-mOTU matrix (*n* = 16 hosts, excluding cattle). We calculated Bray–Curtis dissimilarity and UniFrac distance matrices and used perMANOVA to test the effects of log-transformed body size, group size, understorey foraging and digestive strategy.

We performed a Procrustes cophylogenetic analysis [61] using *paco* [62]. We calculated a goodness-of-fit statistic (*m*^2^*_XY_*) for the data and 1000 random permutations to assess whether observed links between hosts and parasites showed greater phylogenetic congruence (cophylogeny) than expected by chance.

#### (iii) Parasite sharing and host centrality

We built a bipartite species-level network for host species (*n* = 16, including cattle but excluding kudu, for which only three samples had metabarcoding data; electronic supplementary material, table S1). Because parasite-sharing networks may be sensitive to low-abundance links (e.g. arising from consumption and passage of eggs and larvae that do not infect the host), we excluded associations when mean RRA across individuals of a species for a given mOTU was less than 2% (see electronic supplementary material, figure S2 and appendix III for sensitivity analyses of RRA thresholds). To investigate the distribution of parasite host breadth (i.e. the number of host species infected by each parasite mOTU), we compared Akaike's information criterion (AIC) for increasingly aggregated (right-skewed) distributions fitted to the data by maximum-likelihood (*fitdistrplus* [63]). Aggregated distributions indicate many specialists and few generalists.

We projected the network to a unipartite host species network, weighting edges using the sum product of RRA values for shared mOTUs. We calculated degree, closeness, eigenvector, and betweenness centralities for host species using *igraph* [64]. We fitted phylogenetic generalized least-squares models (PGLS) (*caper* [65]) of host node metrics using log-transformed body mass, social-group size, understorey diet, digestive strategy and evolutionary distinctiveness (evol.distinct in *picante* [54] using the ‘fair proportions’ method [66]) as predictors.

## 3. Results

### (a) Influence of host identity and traits on parasite prevalence and diversity

**Figure 1.**
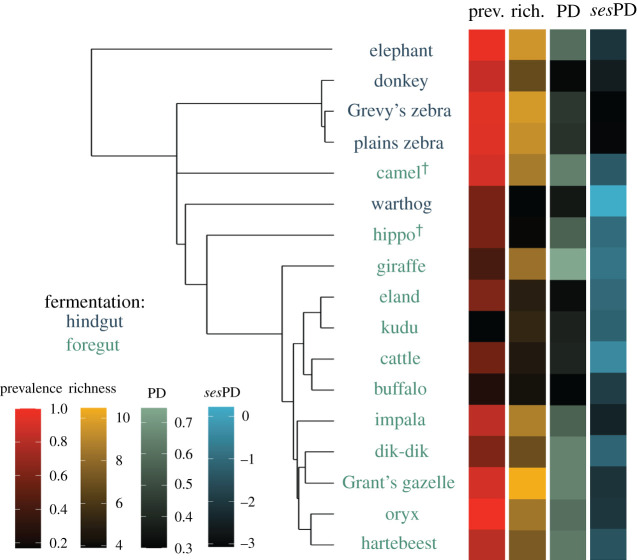
Mean prevalence and diversity metrics across the host phylogeny and different digestion types (see also boxplots in electronic supplementary material, figure S5); † denotes that camel and hippo are foregut-fermenting pseudoruminants, and are distinct from other foregut fermenters (all true ruminants). Total prevalence trended higher and standardized PD (*ses*PD) trended lower in hindgut fermenters compared to foregut fermenters, although these effects were not statistically significant after controlling for phylogenetic relatedness (electronic supplementary material, table S4). We found no significant effect of digestive strategy for richness or phylogenetic diversity (Faith's PD). (Online version in colour.)

We detected 80 parasite mOTUs across 281 sequenced and filtered samples (mean = 15 868 reads per sample). Total prevalence and mean richness were positively correlated (*ρ* = 0.75, *p =* 0.0005; electronic supplementary material, table S1). Host species identity accounted for 27% of variance in total prevalence (*n* = 519, $\chi_{16}^{2}=92.94,$ *p* < 0.001) and 48% of variation in mOTU richness (*n* = 281, $\chi_{16}^{2}=164.29,$ *p* < 0.001). Oryx, elephant, Grevy's and plains zebras, and Grant's gazelle had high total prevalence (all greater than or equal to 0.90) and mean richness (all greater than or equal to 8.44), while kudu, buffalo, cattle, warthog and hippo had relatively low prevalence and richness (electronic supplementary material, table S1 and figure S4). Parasite phylogenetic diversity also differed significantly among host species (PD: *R*^2^ = 0.21, $\chi_{16}^{2}=67.70,$ *p* < 0.001; *ses*PD: *R*^2^ = 0.42, $\chi_{16}^{2}=181.57,$ *p* < 0.001). Cattle and warthog had low prevalence and richness but higher PD after controlling for richness (*ses*PD); giraffe had high richness and PD. At the other end of the spectrum, plains and Grevy's zebras had higher richness but lower *ses*PD (figure 1; electronic supplementary material, table S1 and figure S4).

Although total prevalence and richness trended higher in hindgut fermenters than foregut fermenters, no-host trait was significantly associated with prevalence or richness after accounting for phylogeny (figure 1; electronic supplementary material, table S4). Similarly, while parasite *ses*PD trended lower in hindgut fermenters, this association was non-significant after accounting for host phylogeny, and no other species-level characteristic explained significant variation in PD or *ses*PD (electronic supplementary material, table S4).

### (b) Host species identity and gut anatomy predict parasite community composition

**Figure 2.**
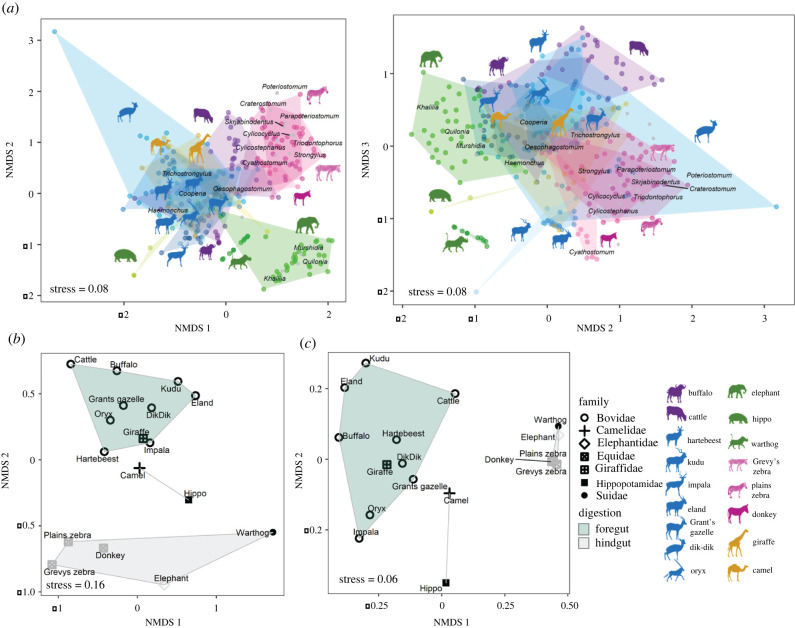
(*a*) Non-metric multidimensional scaling (NMDS) based on Bray–Curtis dissimilarities (*k* = 3 dimensions) for all samples qPCR-positive for nematode DNA; plots show ordinations of the 1st and 2nd (left) and 2nd and 3rd (right) NMDS axes. Polygons are shaded by host species and parasite genera are plotted by mean ordination value. (*b*,*c*) Ordination plots using (*b*) Bray–Curtis dissimilarities for the host species-by-mOTU matrix and (*c*) weighted UniFrac distances for each species demonstrate partitions based on digestive strategy and host taxonomy. (Online version in colour.)

Host species identity strongly predicted individual-level parasite community structure, explaining 53% of compositional dissimilarity variance (perMANOVA *F*_16,264_ = 18.48, *p* < 0.001; figure 2*a*) and 53% of parasite phylogenetic beta-diversity variance (UniFrac distances) among individuals (perMANOVA *F*_16,264_ = 18.82, *p* < 0.001). In the six broadly sampled host species, species identity had far greater explanatory power than time period when animals were considered together ($R_{\mathrm{species}}^{2}=0.49,$ *p* = 0.001, $R_{\mathrm{period}}^{2}=0.02,$ *p* = 0.28), and period was nonsignificant for five of six species when analysed separately, with elephants being the exception (electronic supplementary material, figure S6). We found significant congruence between host and parasite phylogenies (asymmetric *m*^2^*_XY_* = 1 741 295, symmetric *m*^2^*_XY_* = 0.911, both *p* < 0.001 with 1000 permutations).

Parasite communities segregated based on host digestive type (figure 2; electronic supplementary material, table S5): foregut fermenters separated strongly from hindgut fermenters. Two foregut-fermenting pseudoruminants (hippo and camel) were outliers relative to true ruminants (figure 2). Broadly speaking, hindgut fermenters had higher mean RRA of Strongylidae, while foregut fermenters had higher RRA of Cooperiidae, Haemonchidae and Trichostrongylidae (electronic supplementary material, figure S8). While digestive strategy and taxonomic order (a proxy for phylogeny) are closely related and explained similar variance in parasite composition, we found that a model with both predictors had the greatest explanatory power ($R_{\mathrm{Digestion}}^{2}=0.21,$ $R_{\mathrm{Order}}^{2}=0.32,$ *R*^2^_Order+Digestion_ = 0.43). Digestive strategy had an even stronger influence on UniFrac distances, which accounts for mOTU similarity ($R_{\mathrm{Digestion}}^{2}=0.54$, $R_{\mathrm{Order}}^{2}=0.42$, *R*^2^_Order+Digestion_= 0.58). This is visualized in figure 2*c*: hindgut-fermenting species from multiple families cluster closely together. We found no evidence that body size, social-group size, or understorey foraging had significant effects on parasite community composition (electronic supplementary material, table S5).

### (c) Parasite host specificity and parasite-sharing patterns

In our network of host–parasite connections (filtered to mean RRA greater than 2% at the species level), parasite–host breadth was highly aggregated (right-skewed) and followed a lognormal distribution (electronic supplementary material, table S6), indicating many host specialists and few generalists, as predicted. Host breadth varied among parasite families (1–10, median 2), but 47% of mOTUs infected just one species and only 25% infected greater than 3 (electronic supplementary material, figure S7). The most generalized parasite (10 hosts) was identified (98% bootstrap identity score) as *Cooperia fuelleborni*, and was detected in all foregut fermenters except cattle and hartebeest.

**Figure 3.**
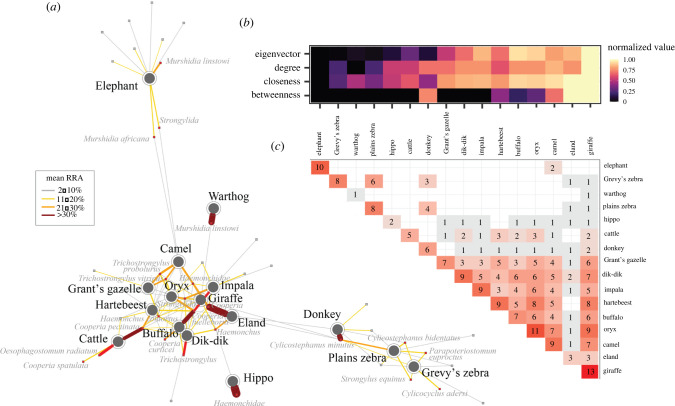
Parasite-sharing networks. (*a*) Bipartite sharing matrix showing large herbivores connected to parasites. Host–parasite edges comprising greater than or equal to 2% mean RRA per host species are weighted by mean RRA. Nodes are plotted using the force-directed algorithm [67]. (*b*) Centrality metrics for host species, normalized to (0,1) and ordered from left to right by increasing combined centrality scores (sum of normalized metrics). (*c*) Weighted adjacency matrix from a unipartite projection of (*a*) shows the number of mOTUs shared between each pair of species; diagonal edge represents the number of mOTUs found with greater than or equal to 2% mean RRA per species (note that this differs from total mOTU richness in electronic supplementary material, table S1, which is filtered at the individual level, rather than species level). (Online version in colour.)

Network node metrics suggested that giraffe, eland and camel may be important species in the parasite-sharing network, as they exhibited relatively high centrality scores across metrics (figure 3*c*). Closeness and eigenvector centralities were higher in foregut fermenters after controlling for phylogenetic relatedness, and we found no evidence for patterns in network centrality associated with other host characteristics (electronic supplementary material, table S7). Evolutionary distinctiveness was not predictive of network centrality metrics in PGLS models because it correlated with phylogenetic signal. When considered separately, degree and closeness centrality metrics were negatively correlated with host evolutionary distinctiveness, and eigenvector centrality trended similarly (electronic supplementary material, figure S9). Bovids were highly interconnected, each sharing parasites with 8–14 other hosts (figure 3*a,b*). Notably, cattle shared parasites with 8 other hosts, whereas elephant and warthog each shared just one mOTU with another species (figure 3*a,b*; electronic supplementary material, Appendix IV).

## 4. Discussion

### (a) Host relatedness and digestive strategy structure parasite communities

Our analysis revealed powerful signals of host species identity in determining parasite mOTU prevalence, richness and phylogenetic diversity, with strong evidence of host–parasite cophylogeny. This cophylogenetic signal is consistent with findings in similar host–parasite systems [20,22] and host–microbiome associations [43,68]. Zebras had high mOTU richness [18], and equids are known to be infected by a diverse array of ‘small strongyles’ [69]; indeed hindgut fermenters in general were highly parasitized by this family (Strongylidae). However, Grant's gazelle and elephant had comparable mOTU richness to zebras; we have no ready explanation for this finding, as these species are phylogenetically and ecologically disparate. Further research that incorporates information about host immunity and parasite natural history may provide more mechanistic insight.

Consistent with previous work comparing ruminants and equids in Botswana [18], we found that parasite prevalence in hindgut-fermenting herbivores trended higher than foregut fermenters, although this difference was not significant after accounting for phylogenetic relatedness. Hindgut fermenters consume large quantities of vegetation to meet energy requirements and thus may have greater exposure to parasitic larvae, leading to higher infection probability. However, this interpretation should also apply to large versus small-bodied species, which also differ in total biomass consumption [70], yet we found no effect of body size on any response. Phylogeny and digestive strategy explained similar variation in standardized phylogenetic diversity: with the exception of warthog, nematodes of hindgut fermenters tended to have lower *ses*PD, which might stem from simpler gut architectures (fewer parasite niches) and/or cophylogenetic constraints on parasite diversification and persistence (perissodactyls and proboscideans are less phylogenetically diverse than artiodactyls, and non-ruminants are a small and shrinking share of east African faunas [71]). Other internal factors (e.g. host immune responses) undoubtedly regulate the relative fitness of parasite taxa and shape diversity and cooccurrence patterns found here [72,73].

Although some previous studies have linked parasite richness with host body size [74], range size [26,75] and/or social-group size [76], none of these traits predicted individual mOTU richness in our study. This is not unprecedented; other studies have failed to detect relationships between richness and body size [77], or group size [75]. These mixed results may be caused by variability in trait values across taxa and differing taxonomic and geographical scales. Population-specific estimates of range size, social-group size (including interspecific groups that feed together [32,78]) and body size may be needed to detect fine-scale effects of these factors on parasite communities. Although we do not have intraspecific host density estimates for all species in this assemblage, we doubt that this factor would explain our results: dik-dik are by far the densest species at Mpala (up to 100 per km^2^ [42]) but had intermediate parasite prevalence and diversity values.

We found that cattle had comparatively high standardized phylogenetic diversity, despite lower parasite prevalence than other species. At our study site, cattle are treated with anthelminthics at weaning and irregularly thereafter, perhaps explaining why cattle had low parasite richness and were free of the most generalized mOTU (putatively *Cooperia fuelleborni*). One hypothesis for the high phylogenetic diversity in cattle is that sporadic anthelminthic treatment may eliminate common and competitively dominant parasite taxa, enabling infection by a wider variety of competing taxa. Indeed, another study in Amboseli National Park, Kenya, found unexpectedly high parasite richness in livestock, despite likely treatment with anthelminthics [15]. Elsewhere, regular deworming significantly reduces parasite diversity [50], and regular treatment of sheep and goats at Mpala resulted in the complete absence of Strongylida DNA. Other domestic animals in the system (donkeys and camels) were not regularly treated for parasites and had at least 90% prevalence. Given that domestic species shared many parasites with wild ones and are regionally increasing in abundance [79], monitoring and managing cross-infections will be increasingly important for both wildlife conservation and livestock production.

The dissimilarity of parasite communities across host species and lineages and the significant results from our cophylogenetic analysis are consistent with a strong coevolutionary relationship between nematode parasites and their herbivore hosts. These results mirror previous studies, especially on parasites of rodents (e.g. [19,80]). While stratified and intensive sampling across periods may reveal finer within-species patterns of parasite community shifts, our results suggest that Strongylida community structure may be largely predictable from host species composition in African ungulate assemblages, and that the impacts of species losses or invasions might also be predictable.

### (b) Parasite host specificity and parasite sharing patterns

Our finding that host–parasite links were highly aggregated aligns with previous studies showing that most parasites specialize on few hosts while only a few have broad host ranges [18,81]. Parasites with broad host ranges are especially important when considering potential for parasite sharing at the wildlife-livestock interface and for predicting missing links in host–parasite networks [18]. Indeed, we found that evolutionarily distinct species still shared some parasites with central species, and that camels and giraffe had high levels of parasite overlap with bovids (figure 3*a,b*; electronic supplementary material, figure S9), demonstrating the role of a few generalists in connecting the network. The high centrality of giraffe and camel—intriguing given that each is the only local member of its family and that both feed overwhelmingly in the overstorey—suggest that these species are infected by generalist parasites and may play an important role in cross-species transmission depending on their movement and density. One clear management implication is that regularly deworming camels, as done successfully for sheep and goats at our site, might help limit parasite transmission to wildlife (notably giraffe, a threatened species). The same is true for donkeys, which had the highest centrality of the three equids and are likely to share parasites with the endangered Grevy's zebra and near-threatened plains zebra.

Our finding of phylogenetic influence on network centrality metrics is consistent with the likelihood that closely related hosts share ecological, anatomical and immunological traits that shape host–parasite coevolution. Indeed, models based on herbivore parasite records [18] suggest that host–parasite cophylogeny facilitates prediction of parasite-sharing networks. Our results captured similar patterns non-invasively in a single study, showing that the composition of host assemblages can predict attributes of parasite assemblages, including potential patterns of parasite coextinction arising from loss of evolutionarily distinct species.

### (c) Caveats

While the DNA barcode we sequenced can effectively distinguish parasitic nematode species across a wide range of hosts [50], it is likely that parasite species have different ITS-2 copy numbers per cell, different numbers of cells, and perhaps primer mismatches, which can affect amplification success and the accuracy of RRA as an indicator of the relative abundance of parasite species.

Our raw data contained low-abundance mOTUs that generated a highly interconnected host–host sharing network, but filtered data using mean RRA greater than or equal to 2% per species were more concordant with prior studies [12,13]. While sequencing errors, tag jumps, and contamination can contribute to low-abundance mOTUs distributed across samples [82], recent work has stressed the challenge of mitigating such errors while retaining low-abundance mOTUs that are real and important components of ecological networks [83,84]. For gastrointestinal-parasite networks, low-abundance mOTUs could arise from excreted parasites that do not cause infection, or from ‘satellite species’ that produce rare low-intensity infections [85]. Further research combining parasitological work with DNA sequencing will help to optimize methods to enable reliable detection of satellite species and to enhance reference libraries (e.g. [15,86]).

We could not identify 48% of parasite mOTUs to species due to gaps in genetic resources for many wildlife parasites [17]. While these taxa are understudied compared to human and livestock parasites, they may be important for wildlife conservation [18]. Efforts to connect DNA sequences with the taxonomic and natural-history knowledge preserved in collections will provide further insights. For example, knowledge of parasite virulence for each host species could be used to weight edges in host–parasite sharing networks to identify hosts that are central to costly interactions. Although metabarcoding is not a substitute for parasitological expertise, our results provide compelling evidence for its potential to greatly augment our understanding of broad patterns in parasite ecology.

## 5. Conclusion

We show that host phylogeny and digestive strategy explain a high degree of variance in parasite community composition and sharing across a diverse community of wild and domestic large herbivores. While additional work is needed to maximize the value of parasite metabarcoding data, this approach holds enormous promise to shed light beneath the tip of the biodiversity iceberg. Use of these methods in studies with spatially or temporally stratified sampling designs will allow researchers to capture multi-host parasite dynamics that have long remained elusive. Our finding of substantial parasite sharing at the livestock-wildlife interface suggests that regularly deworming camels and donkeys could be an effective local management intervention to reduce transmission between livestock and several globally threatened and near-threatened ungulates. More broadly, our results suggest that changes to domestic animal communities or their parasite loads (e.g. via stocking density, ranging patterns or anthelminthic treatment) will impact parasitism in sympatric wild hosts.

## Data Availability

Data and code are available at Dryad Digital Repository: https://doi.org/10.25349/D96P6K [87]. The data are provided in electronic supplementary material [88].

## References

[1] Lafferty KD, Dobson AP, Kuris AM. 2006 Parasites dominate food web links. Proc. Natl Acad. Sci. USA **103**, 11 211-11 216. (10.1073/pnas.0604755103) PMC1544067 16844774

[2] Poulin R. 1999 The functional importance of parasites in animal communities: many roles at many levels? Int. J. Parasitol. **29**, 903-914. 10480727 10.1016/s0020-7519(99)00045-4

[3] Woolhouse M, Taylor L, Haydon D. 2001 Population biology of multihost pathogens. Science **292**, 1109-1112. 11352066 10.1126/science.1059026

[4] Bordes F, Morand S. 2009 Parasite diversity: an overlooked metric of parasite pressures? Oikos **118**, 801-806. (10.1111/j.1600-0706.2008.17169.x)

[5] Pilosof S, Morand S, Krasnov BR, Nunn CL. 2015 Potential parasite transmission in multi-host networks based on parasite sharing. PLoS ONE **10**, e0117909. (10.1371/journal.pone.0117909) 25748947 PMC4352066

[6] Guernier V, Hochberg ME, Guégan J-F. 2004 Ecology drives the worldwide distribution of human diseases. PLoS Biol. **2**, e141. (10.1371/journal.pbio.0020141) 15208708 PMC423130

[7] Poulin R. 2014 Parasite biodiversity revisited: Frontiers and constraints. Int. J. Parasitol. **44**, 581-589. (10.1016/j.ijpara.2014.02.003) 24607559

[8] Hadfield J. 2010 MCMC Methods for multi-response generalized linear mixed models: the MCMCglmm R package. J. Stat. Softw. **33**, 1-22.20808728

[9] Veldhuis MP, Kihwele ES, Cromsigt JPGM, Ogutu JO, Hopcraft JGC, Owen-Smith N, Olff H. 2019 Large herbivore assemblages in a changing climate: incorporating water dependence and thermoregulation. Ecol. Lett. **22**, 1536-1546. (10.1111/ELE.13350) 31332945 PMC6851681

[10] Dirzo R, Young HS, Galetti M, Ceballos G, Isaac NJB, Collen B. 2014 Defaunation in the Anthropocene. Science **345**, 401-406. (10.1126/science.1251817) 25061202

[11] Bar-On YM, Phillips R, Milo R. 2018 The biomass distribution on Earth. Proc. Natl Acad. Sci. USA **115**, 6506-6511. (10.1073/pnas.1711842115) 29784790 PMC6016768

[12] Nunn CL, Altizer SM. 2005 The global mammal parasite database: An online resource for infectious disease records in wild primates. Evol. Anthropol. Issues, News, Rev. **14**, 1-2. (10.1002/evan.20041)

[13] Gibson DI, Bray RA, Harris EA. 2005 Host-Parasite database of the natural history museum. London, UK: Natural History Museum. See https://www.nhm.ac.uk/research-curation/scientific-resources/taxonomy-systematics/host-parasites/.

[14] Bass D, Stentiford GD, Littlewood DTJ, Hartikainen H. 2015 Diverse applications of environmental DNA methods in parasitology. Trends Parasitol. **31**, 499-513. (10.1016/j.pt.2015.06.013) 26433253

[15] Obanda V, Maingi N, Muchemi G, Ng'Ang'A CJ, Angelone S, Archie EA. 2019 Infection dynamics of gastrointestinal helminths in sympatric non-human primates, livestock and wild ruminants in Kenya. PLoS ONE **14**, e0217929. (10.1371/JOURNAL.PONE.0217929) 31181093 PMC6557494

[16] Titcomb GC, Jerde CL, Young HS. 2019 High-throughput sequencing for understanding the ecology of emerging infectious diseases at the wildlife-human interface. Front. Ecol. Evol. **7**, 126. (10.3389/fevo.2019.00126)

[17] Selbach C et al. 2019 Parasitological research in the molecular age. Parasitology **146**, 1361-1370. (10.1017/S0031182019000726) 31142396

[18] Walker JG, Plein M, Morgan ER, Vesk PA. 2017 Uncertain links in host-parasite networks: lessons for parasite transmission in a multi-host system. Phil. Trans. R. Soc. B **372**, 20160095. (10.1098/rstb.2016.0095) 28289262 PMC5352821

[19] Bordes F, Morand S. 2008 Helminth species diversity of mammals: parasite species richness is a host species attribute. Parasitology **135**, 1701-1705. (10.1017/S0031182008005040) 18992179

[20] Gogarten JF et al. 2020 Metabarcoding of eukaryotic parasite communities describes diverse parasite assemblages spanning the primate phylogeny. Mol. Ecol. Resour. **20**, 204-215. (10.1111/1755-0998.13101) 31600853

[21] Poulin R. 2007 Are there general laws in parasite ecology? Parasitology **134**, 763-776. (10.1017/S0031182006002150) 17234043

[22] Poulin R. 1995 Phylogeny, ecology, and the richness of parasite communities in vertebrates. Ecol. Monogr. **65**, 283-302. (10.2307/2937061)

[23] Morand S. 2015 (macro-) Evolutionary ecology of parasite diversity: from determinants of parasite species richness to host diversification. Int. J. Parasitol. Parasites Wildl. **4**, 80-87. (10.1016/j.ijppaw.2015.01.001) 25830109 PMC4356877

[24] Kamiya T, O'Dwyer K, Nakagawa S, Poulin R. 2014 What determines species richness of parasitic organisms? A meta-analysis across animal, plant and fungal hosts. Biol. Rev. **89**, 123-134. (10.1111/brv.12046) 23782597

[25] Poulin R, Krasnov BR, Mouillot D, Thieltges DW. 2011 The comparative ecology and biogeography of parasites. Phil. Trans. R. Soc. B **366**, 2379-2390. (10.1098/rstb.2011.0048) 21768153 PMC3130428

[26] Lindenfors P, Nunn CL, Jones KE, Cunningham AA, Sechrest W, Gittleman JL. 2007 Parasite species richness in carnivores: effects of host body mass, latitude, geographical range and population density. Glob. Ecol. Biogeogr. **16**, 496-509. (10.1111/j.1466-8238.2006.00301.x)

[27] Ezenwa VO. 2004 Host social behavior and parasitic infection: a multifactorial approach. Behav. Ecol. **15**, 446-454. (10.1093/BEHECO/ARH028)

[28] Rohde K. 1994 Niche restriction in parasites: Proximate and ultimate causes. Parasitology **109**, S69-S84. (10.1017/s0031182000085097) 7854853

[29] Hannon ER, Kinsella JM, Calhoun DM, Joseph MB, Johnson PTJ. 2016 Endohelminths in bird hosts from Northern California and an analysis of the role of life history traits on parasite richness. J. Parasitol. **102**, 199-207. (10.1645/15-867) 26579621 PMC4855505

[30] Marques JF, Santos MJ, Cabral HN, Poulet SA, Marques RJF, Cabral HN, Santos MJ. 2006 Soleidae macroparasites along the Portuguese coast: latitudinal variation and host-parasite associations. Mar. Biol. **150**, 285-298. (10.1007/s00227-006-0339-8)

[31] Titcomb G, Mantas JN, Hulke J, Rodriguez I, Branch D, Young H. 2021 Water sources aggregate parasites with increasing effects in more arid conditions. Nat. Commun. **121**, 1-12. (10.1038/s41467-021-27352-y) PMC8642388 34862389

[32] VanderWaal K, Omondi GP, Obanda V. 2014 Mixed-host aggregations and helminth parasite sharing in an East African wildlife-livestock system. Vet. Parasitol. **205**, 224-232. (10.1016/j.vetpar.2014.07.015) 25086496

[33] Lafferty KD. 2012 Biodiversity loss decreases parasite diversity: theory and patterns. Phil. Trans. R. Soc. B **367**, 2814-2827. (10.1098/rstb.2012.0110) 22966137 PMC3427564

[34] Treonis AM, Unangst SK, Kepler RM, Buyer JS, Cavigelli MA, Mirsky SB, Maul JE. 2018 Characterization of soil nematode communities in three cropping systems through morphological and DNA metabarcoding approaches. Sci. Rep. **81**, 1-12. (10.1038/s41598-018-20366-5) PMC5792604 29386563

[35] Tombak KJ, Hansen CB, Kinsella JM, Pansu J, Pringle RM, Rubenstein DI. 2021 The gastrointestinal nematodes of plains and Grevy's zebras: phylogenetic relationships and host specificity. Int. J. Parasitol. Parasites Wildl. **16**, 228-235. (10.1016/J.IJPPAW.2021.10.007) 34712556 PMC8529100

[36] Davey ML, Utaaker KS, Fossøy F. 2021 Characterizing parasitic nematode faunas in faeces and soil using DNA metabarcoding. Parasites Vectors **14**, 1-13. (10.1186/S13071-021-04935-8/FIGURES/3) 34419166 PMC8380370

[37] Poissant J et al. 2021 A repeatable and quantitative DNA metabarcoding assay to characterize mixed strongyle infections in horses. Int. J. Parasitol. **51**, 183-192. (10.1016/J.IJPARA.2020.09.003) 33242465

[38] Crofton HD. 1948 The ecology of immature phases of trichostrongyle nematodes: I. the vertical distribution of infective larvae of trichostrongylus retortaeformis in relation to their habitat. Parasitology **39**, 17-25. (10.1017/S0031182000083517) 18876873

[39] Shaw DJ, Grenfell BT, Dobson AP. 1998 Patterns of macroparasite aggregation in wildlife host populations. Parasitology **117**, 597-610. 9881385 10.1017/s0031182098003448

[40] Dallas T, Han B, Nunn C, Park A, Stephens P, Drake J. 2019 Host traits associated with species roles in parasite sharing networks. Oikos **128**, 23-32. (10.1111/oik.05602)

[41] Goheen JR, Palmer TM, Charles GK, Helgen KM, Kinyua SN, Maclean JE, Turner BL, Young HS, Pringle RM. 2013 Piecewise disassembly of a large-herbivore community across a rainfall gradient: the UHURU experiment. PLoS ONE **8**, e55192. (10.1371/journal.pone.0055192) 23405122 PMC3566220

[42] Alston J et al. 2021 Ecological consequences of large-herbivore exclusion in an African savanna: 12 years of data from the UHURU experiment. Ecology **103**. (10.1002/ECY.3649) 35084743

[43] Kartzinel TR, Hsing JC, Musili PM, Brown BRP, Pringle RM. 2019 Covariation of diet and gut microbiome in African megafauna. Proc. Natl Acad. Sci. USA **116**, 23 588-23 593. (10.1073/PNAS.1905666116) 31685619 PMC6876249

[44] Augus7tine DJ. 2010 Response of native ungulates to drought in semi-arid Kenyan rangeland. Afr. J. Ecol. **48**, 1009-1020. (10.1111/j.1365-2028.2010.01207.x)

[45] Kartzinel TR, Pringle RM. 2020 Multiple dimensions of dietary diversity in large mammalian herbivores. J. Anim. Ecol. **89**, 1482-1496. (10.1111/1365-2656.13206) 32163591

[46] IUCN. 2021 The IUCN Red List of Threatened Species. Version 2021-2. See https://www.iucnredlist.org.

[47] Gasser RB, Chilton NB, Hoste H, Beveridge I. 1993 Rapid sequencing of rDNA from single worms and eggs of parasitic helminths. Nucleic Acids Res. **21**, 2525. (10.1093/NAR/21.10.2525) 8506152 PMC309567

[48] Zajac A, Conboy GA. 2012 Fecal examination for the diagnosis of parasitism. In Veterinary clinical parasitology (eds AM Zajac, GA Conboy), pp. 3-171. Chichester, UK: Wiley-Blackwell.

[49] Boyer F, Mercier C, Bonin A, Le Bras Y, Taberlet P, Coissac E. 2016 obitools: a unix-inspired software package for DNA metabarcoding. Mol. Ecol. Resour. **16**, 176-182. 25959493 10.1111/1755-0998.12428

[50] Avramenko RW, Redman EM, Lewis R, Bichuette MA, Palmeira BM, Yazwinski TA, Gilleard JS. 2017 The use of nemabiome metabarcoding to explore gastro-intestinal nematode species diversity and anthelmintic treatment effectiveness in beef calves. Int. J. Parasitol. **47**, 893-902. (10.1016/J.IJPARA.2017.06.006) 28797791

[51] McK7night DT, Huerlimann R, Bower DS, Schwarzkopf L, Alford RA, Zenger KR. 2019 microDecon: a highly accurate read-subtraction tool for the post-sequencing removal of contamination in metabarcoding studies. Environ. DNA **1**, 14-25. (10.1002/EDN3.11)

[52] Cameron ES, Schmidt PJ, Tremblay BJM, Emelko MB, Müller KM. 2021 Enhancing diversity analysis by repeatedly rarefying next generation sequencing data describing microbial communities. Sci. Rep. **111**, 1-13. (10.1038/s41598-021-01636-1) PMC8595385 34785722

[53] R Core Team. 2016 R: a language and environment for statistical computing. Vienna, Austria: R Foundation for Statistical Computing.

[54] Kembel SW, Cowan PD, Helmus MR, Cornwell WK, Morlon H, Ackerly DD, Blomberg SP, Webb CO. 2010 Picante: R tools for integrating phylogenies and ecology. Bioinformatics **26**, 1463-1464. (10.1093/bioinformatics/btq166) 20395285

[55] Molina-Venegas R. 2019 Avoiding potential biases in ses.PD estimations with the Picante software package. bioRxiv 579300. (10.1101/579300)

[56] Upham NS, Esselstyn JA, Jetz W. 2019 Inferring the mammal tree: Species-level sets of phylogenies for questions in ecology, evolution, and conservation. PLoS Biol. **17**, e3000494. (10.1371/JOURNAL.PBIO.3000494) 31800571 PMC6892540

[57] Jon7es KE et al. 2009 PanTHERIA: a species-level database of life history, ecology, and geography of extant and recently extinct mammals. Ecology **90**, 2648. (10.1890/08-1494.1)

[58] Oksanen J et al. 2016 vegan: Community Ecology Package.

[59] Lozupone C, Knight R. 2005 UniFrac: a new phylogenetic method for comparing microbial communities. Appl. Environ. Microbiol. **71**, 8228-8235. (10.1128/AEM.71.12.8228-8235.2005) 16332807 PMC1317376

[60] McMurdie PJ, Holmes S. 2013 phyloseq: an R package for reproducible interactive analysis and graphics of microbiome census data. PLoS ONE **8**, e61217. (10.1371/journal.pone.0061217) 23630581 PMC3632530

[61] Balbuena JA, Míguez-Lozano R, Blasco-Costa I. 2013 PACo: A novel Procrustes application to cophylogenetic analysis. PLoS ONE **8**, e61048. (10.1371/journal.pone.0061048) 23580325 PMC3620278

[62] Hutchinson MC, Cagua EF, Balbuena JA, Stouffer DB, Poisot T. 2017 paco: implementing Procrustean approach to cophylogeny in R. Methods Ecol. Evol. **8**, 932-940. (10.1111/2041-210X.12736)

[63] Delignette-Muller ML, Dutang C. 2015 fitdistrplus: an R package for fitting distributions. J. Stat. Softw. **64**, 1-34. (10.18637/JSS.V064.I04)

[64] Csardi G, Nepusz T. 2006 The igraph software package for complex network research. InterJournal Complex Systems **1695**. See https://igraph.org.

[65] Orme D, Freckleton R, Thomas G, Petzoldt T, Fritz S, Isaac N, Pearse W. 2018 The caper package: comparative analysis of phylogenetics and evolution in R.

[66] Isaac NJB, Turvey ST, Collen B, Waterman C, Baillie JEM. 2007 Mammals on the EDGE: conservation priorities based on threat and phylogeny. PLoS ONE **2**, e296. (10.1371/JOURNAL.PONE.0000296) 17375184 PMC1808424

[67] Fruchterman TMJ, Reingold EM. 1991 Graph drawing by force-directed placement. Softw. Pract. Exp. **21**, 1129-1164. (10.1002/SPE.4380211102)

[68] Lim SJ, Bordenstein SR. 2020 An introduction to phylosymbiosis. Proc. R. Soc. B **287**, 20192900. (10.1098/RSPB.2019.2900) PMC7126058 32126958

[69] Corning S. 2009 Equine cyathostomins: a review of biology, clinical significance and therapy. Parasites Vectors **2**, S1. (10.1186/1756-3305-2-S2-S1) PMC2751837 19778462

[70] Clauss M, Steuer P, Müller DWH, Codron D, Hummel J. 2013 Herbivory and body size: allometries of diet quality and gastrointestinal physiology, and implications for herbivore ecology and dinosaur gigantism. PLoS ONE **8**, e68714. (10.1371/JOURNAL.PONE.0068714) 24204552 PMC3812987

[71] Faith J T, Rowan J, Du A. 2019 Early hominins evolved within non-analog ecosystems. Proc. Natl Acad. Sci. USA **116**, 21 478-21 483. (10.1073/PNAS.1909284116/-/DCSUPPLEMENTAL) PMC6815188 31591246

[72] Bashey F. 2015 Within-host competitive interactions as a mechanism for the maintenance of parasite diversity. Phil. Trans. R. Soc. B **370**, 20140301. (10.1098/RSTB.2014.0301) 26150667 PMC4528499

[73] Chesson P. 2000 Mechanisms of maintenance of species diversity. Annu. Rev. Ecolo **31**, 343-366. (10.1146/ANNUREV.ECOLSYS.31.1.343)

[74] Ezenwa VO, Price SA, Altizer S, Vitone ND, Cook KC. 2006 Host traits and parasite species richness in even and odd-toed hoofed mammals, Artiodactyla and Perissodactyla. Oikos **115**, 526-536. (10.1111/j.2006.0030-1299.15186.x)

[75] Nunn CL, Altizer S, Jones KE, Sechrest W. 2003 Comparative Tests of Parasite Species Richness in Primates. Am. Nat. **162**, 597-614. (10.1086/378721) 14618538

[76] Ezenwa VO, Worsley-Tonks KEL. 2018 Social living simultaneously increases infection risk and decreases the cost of infection. Proc. R. Soc. B **285**, 20182142. (10.1098/RSPB.2018.2142) PMC6283948 30487314

[77] Morand S, Poulin R. 1998 Density, body mass and parasite species richness of terrestrial mammals. Evol. Ecol. **12**, 717-727. (10.1023/A:1006537600093)

[78] Ezenwa VO. 2003 Habitat overlap and gastrointestinal parasitism in sympatric African bovids. Parasitology **126**, 379-388. (10.1017/S0031182002002913) 12741517

[79] Ogutu JO, Piepho H-P, Said MY, Ojwang GO, Njino LW, Kifugo SC, Wargute PW. 2016 Extreme wildlife declines and concurrent increase in livestock numbers in Kenya: what are the causes? PLoS ONE **11**, e0163249. (10.1371/journal.pone.0163249) 27676077 PMC5039022

[80] Dallas T, Presley S. 2014 Relative importance of host environment, transmission potential and host phylogeny to the structure of parasite metacommunities. Oikos **123**, 866-874. (10.1111/oik.00707)

[81] Park AW et al. 2018 Characterizing the phylogenetic specialism–generalism spectrum of mammal parasites. Proc. R. Soc. B **285**, 20172613. (10.1098/RSPB.2017.2613) PMC5879627 29514973

[82] Ando H, Mukai H, Komura T, Dewi T, Ando M, Isagi Y. 2020 Methodological trends and perspectives of animal dietary studies by noninvasive fecal DNA metabarcoding. Environ. DNA **2**, 391-406.

[83] Deagle BE, Thomas AC, McInnes JC, Clarke LJ, Vesterinen EJ, Clare EL, Kartzinel TR, Eveson JP. 2019 Counting with DNA in metabarcoding studies: how should we convert sequence reads to dietary data? Mol. Ecol. **28**, 391-406. (10.1111/mec.14734) 29858539 PMC6905394

[84] Pringle RM, Hutchinson MC. 2020 Resolving food-web structure. Annu. Rev. Ecol. Evol. Syst. **51**, 55-80. (10.1146/ANNUREV-ECOLSYS-110218-024908)

[85] Bush AO, Holmes JC. 2011 Intestinal helminths of lesser scaup ducks: patterns of association. Can. J. Zool. **64**, 132-141. (10.1139/Z86-022)

[86] McLean ER, Kinsella JM, Chiyo P, Obanda V, Moss C, Archie EA. 2012 Genetic identification of five strongyle nematode parasites in wild African elephants (*Loxodonta africana*). J. Wildl. Dis. **48**, 707-716. (10.7589/0090-3558-48.3.707) 22740536

[87] Titcomb GC, Pansu J, Hutchinson MC, Tombak KJ, Hansen CB, Baker CCM, Kartzinel TR, Young HS, Pringle RM. 2022 Data from: Large-herbivore nemabiomes: Patterns of parasite diversity and sharing. *Dryad Digital Repository.* (10.25349/D96P6K) PMC9091847 35538775

[88] Titcomb GC, Pansu J, Hutchinson MC, Tombak KJ, Hansen CB, Baker CCM, Kartzinel TR, Young HS, Pringle RM 2022 Large-herbivore nemabiomes: patterns of parasite diversity and sharing. *FigShare*. (10.6084/m9.figshare.c.5958637) PMC9091847 35538775

